# Cold Tolerance in Alfalfa: Physiological Mechanisms, Regulatory Networks, and Molecular Breeding Prospects

**DOI:** 10.3390/plants15203110

**Published:** 2026-10-11

**Authors:** Weiguang Yang, Xiaojia Shi, Jinyang Lu, Xiangping Liu

**Affiliations:** College of Animal Science and Technology, Heilongjiang Bayi Agricultural University, Daqing 163319, China; yangweiguang2021@byau.edu.cn (W.Y.); shixiaojia15731969113@byau.edu.cn (X.S.)

**Keywords:** alfalfa, cold acclimation, freezing tolerance, winter hardiness, root crown, phenotyping, genomic selection

## Abstract

Alfalfa (*Medicago sativa* L.) is a perennial legume forage whose winter survival determines stand persistence, spring green-up, and long-term yield in cold regions. Low-temperature stress causes membrane phase transitions, cellular dehydration, impaired photosynthetic electron transport, and excessive reactive oxygen species (ROS) accumulation. Tolerance depends on coordinated antioxidant protection, osmotic adjustment, membrane stabilization, photosynthetic maintenance, and carbon–nitrogen allocation to the root crown. At the molecular level, this process is mediated by calcium ion signaling, phytohormone pathways, and the cold-responsive ICE–CBF/DREB–COR regulatory cascade. Reported molecular resources include calmodulin-like protein modules (MsCML10 and MsCML50), CBF/DREB factors, autophagy and circadian regulators, sugar-metabolism genes, and genes from yellow-flowered alfalfa. This review integrates low-temperature injury, physiological responses, molecular regulatory networks, evaluation methods, genetic resources, and breeding progress. It emphasizes that evidence strength differs among cultivated-alfalfa functional studies, heterologous validation, and omics-based candidate discovery. Major gaps include inconsistent phenotyping, insufficient root-crown and overwintering-bud characterization, limited validation under natural overwintering, and weak translation of candidate genes into breeding populations. Future research should combine standardized multi-environment phenotyping with multi-omics, functional validation in alfalfa, marker development, genomic selection, and multi-trait improvement of cold tolerance, yield, forage quality, and stress adaptation.

## 1. Introduction

Alfalfa is an important crop for restructuring grassland-based agriculture, securing a supply of high-quality forage, and supporting the high-quality development of animal husbandry in China. Registered alfalfa cultivars in China include bred cultivars, local cultivars, wild-derived cultivated materials, and introduced cultivars. Cold-tolerant cultivars adapted to Northeast China, Inner Mongolia, Northwest China, and alpine regions remain a priority for breeding and regional deployment [1].

Plant adaptation to low temperature generally involves a sequence of cold perception, a decrease in membrane fluidity, activation of calcium-ion (Ca^2+^) signaling, dynamic regulation of reactive oxygen species (ROS), transcription factor cascades, and metabolic reprogramming. Cold tolerance in alfalfa should therefore not be regarded as a single overwintering phenotype, but as a complex quantitative trait determined by cellular protection, organ survival, stand recovery, and genetic background [2]. In production systems, overwintering failure is rarely caused by low temperature alone. It commonly results from the combined effects of extreme cold, freeze–thaw cycles, insufficient snow cover, unstable spring warming, and interactions among cold, drought, and salinity. Mechanistically, low-temperature injury is driven by sustained disruption of cellular water status, membrane structure, redox homeostasis, and metabolic allocation [3]. In regions exposed to combined cold, drought, and salinity, low temperature can also impair rhizobial symbiotic nitrogen fixation, soil water and salt movement, and post-winter plant recovery. Research on alfalfa cold tolerance should therefore integrate cellular tolerance mechanisms, protection of the root crown and overwintering buds, and field-level overwintering performance [4,5].

In this review, cold tolerance is used as the broad capacity to maintain function under non-freezing or freezing low temperatures, whereas freezing tolerance refers specifically to survival of subzero exposure and freeze-induced cellular dehydration. Cold acclimation is the inducible increase in freezing tolerance after prior exposure to low, non-freezing temperatures. Winter hardiness is the integrated capacity of a perennial stand to withstand the full winter complex, including freezing, freeze–thaw events, desiccation, ice encasement, pathogens, and unstable snow cover. Overwintering survival is the field outcome measured after winter and therefore combines genetic winter hardiness with site, year, stand age, and management effects. These terms are related but are not interchangeable [6].

Accordingly, this review focuses on cold tolerance mechanisms and molecular breeding for cold resistance in alfalfa. It summarizes the morphological, physiological, and molecular effects of low-temperature stress; physiological mechanisms involving antioxidant defense, osmotic adjustment, membrane stability, photosynthesis, and primary metabolism; and molecular pathways involving Ca^2+^ signaling, inducer of CBF expression–C-repeat binding factor/dehydration-responsive element-binding–cold-responsive (ICE–CBF/DREB–COR) regulation, phytohormone signaling, autophagy, circadian rhythms, and sugar metabolism. Cold-tolerance evaluation systems, genetic resources, molecular breeding progress, and differences between research in China and other countries are further integrated to identify priorities for future research and breeding applications.

### Literature Search Strategy

This is a narrative, evidence-weighted review rather than a systematic review or meta-analysis. The revision used targeted web-based searches of PubMed/PMC, journal and publisher websites, and official cultivar-registration or institutional sources, together with citation tracing from the existing bibliography. Search concepts combined alfalfa, *Medicago sativa*, or *Medicago falcata* with cold, chilling, freezing, acclimation, winter hardiness, overwintering, root crown, ROS, Ca^2+^, ABA, CBF/DREB, autophagy, circadian regulation, QTL, GWAS, genomic selection, phenomics, multi-omics, and non-coding RNA. Coverage focused on 2010–2026, retaining earlier foundational studies and relevant Chinese-language reports. The final targeted update was completed on 30 September 2026. Studies were distinguished by donor species, validation host, sampled organ, stress regime, and survival endpoint; cultivar catalogues and official notices were used only for claims explicitly documented in those sources. No claim of exhaustive database coverage is made.

## 2. Effects of Low-Temperature Stress on Alfalfa

### 2.1. Types of Low-Temperature Injury and Production Impacts

Low-temperature injury in alfalfa can be classified as chilling injury, freezing injury, freeze–thaw injury, and late-spring cold injury. Chilling injury generally occurs at temperatures above 0 °C. Although intracellular ice does not form, chloroplast function, root absorption, and membrane fluidity are inhibited, resulting in leaf chlorosis, wilting, growth arrest, and delayed green-up. Freezing injury occurs below 0 °C, when extracellular ice formation lowers water potential and causes protoplasmic dehydration. Under rapid or severe cooling, intracellular ice crystals may directly damage organelles and membrane systems. Repeated freezing and thawing causes recurrent contraction and expansion of the root crown and membranes, which can lead to crown cracking, taproot browning, lateral root death, and reduced viability of overwintering buds. Late-spring cold injury occurs after green-up has begun; damage to newly emerging buds, young leaves, and regrowing shoots directly reduces first-cut yield.

Natural snow cover can buffer soil-surface temperature fluctuations, but winter survival still differs among cultivars and fall-dormancy classes [7]. Final harvest timing and cold soda-saline–alkali conditions also influence crown physiology and survival [8,9]. These management and environmental comparisons should not be summarized as a uniform increase or decrease in antioxidant activity or reserve concentration: the direction depends on treatment, genotype, organ, and sampling date. Their practical value is to identify the conditions under which crown protection and spring recovery are maintained, rather than to establish a single universal biochemical mechanism.The main types of low-temperature injury, their symptoms, and corresponding evaluation criteria are summarized in Table 1.

**Table 1:** Types of low-temperature injury in alfalfa, major symptoms, and key evaluation criteria.

Injury Type	Occurrence Conditions	Major Symptoms	Key Evaluation Indicators	Applicable Scenarios
Chilling injury	Sustained low temperature above 0 °C or cold-air events	Leaf chlorosis, wilting, growth arrest, and delayed green-up	Chlorophyll content, Fv/Fm, net photosynthetic rate, and soluble sugars	Seedling screening and spring green-up risk assessment
Freezing injury	Freezing and prolonged low temperature below 0 °C	Cellular dehydration, membrane damage, root-crown browning, and death of overwintering buds	Relative electrolyte conductivity, MDA, LT_50_, and root-crown viability	Controlled freezing-tolerance screening; requires field winter-survival validation
Freeze–thaw injury	Repeated freezing and thawing within a day or across seasons	Root-crown cracking, taproot and lateral-root injury, and stand thinning	Root-crown injury rating, number of overwintering buds, and number of regrowing shoots	Natural and simulated overwintering trials
Late-spring cold injury	Rapid temperature decline after green-up	Necrosis of new buds, reduced regrowth, and lower first-cut yield	Green-up rate, cold-injury rating, first-cut yield, and stand cover	Field risk assessment in spring

### 2.2. Physiological Responses

At the physiological level, low temperature shifts membrane lipids toward a more ordered state, decreases membrane fluidity, disrupts membrane-protein function, and generally increases electrolyte leakage. Freezing injury is therefore commonly accompanied by higher relative electrolyte conductivity, higher malondialdehyde (MDA), tissue browning, and lower root-crown viability. Photosynthetic electron transport is inhibited, while reactive oxygen species (ROS) can rise above the signaling range and promote lipid, protein, and nucleic-acid oxidation. During abrupt chilling, soluble sugars and freezing tolerance can increase during acclimation but decline when injury exceeds metabolic capacity; the direction must therefore be reported together with genotype, organ, temperature, and sampling time [10].

Recent paired-cultivar freezing analysis provides a directional example: compared with the sensitive cultivar Bara 218TR, the cold-tolerant Dongnong No. 1 maintained higher APX, GR, and CAT activities and accumulated more proline and soluble sugars, while showing lower MDA and relative electrolyte leakage under freezing treatment [11]. These variables are useful injury or response indicators, but association with survival does not by itself establish that the measured change causes tolerance. Causal evidence requires genetic or pharmacological perturbation, complementation or rescue, and an organ- or whole-plant survival endpoint. Root studies likewise link higher soluble sugars, proline, glutathione, ABA, JA, and POD activity with tolerant genotypes, while appropriately treating co-expression as candidate evidence rather than functional proof [12].

### 2.3. Molecular Responses

Alfalfa cold responses involve Ca^2+^ transients, redox and hormone signaling, transcriptional regulation, and protective metabolism. Transcriptomic comparisons identify cold-responsive candidates but do not by themselves demonstrate causality [13,14]. Under the phosphate regimes examined by Wang et al., moderate phosphate supply increased soluble sugar and soluble protein contents during acclimation relative to phosphate-deficient plants; integrated transcriptomic and metabolomic data implicated several metabolic networks [15]. This directional result is specific to the tested nutrition and temperature regime and should not be generalized to every metabolite or fertilizer dose.

Transcriptomic studies of roots and root crowns during overwintering suggest that belowground low-temperature responses are associated with subsequent survival and green-up [16]. The root crown is the perennial transition zone that stores reserves and contains overwintering buds; damage at this site can disconnect apparently tolerant leaf physiology from field persistence. It should therefore be a central sampling organ, with autumn acclimation, non-structural carbohydrate and nitrogen reserves, bud viability, winter injury, spring shoot emergence, and first-cut regrowth measured as a linked sequence rather than as isolated endpoints.

### 2.4. Cold-Adapted Germplasm and Cultivars as the Biological Context

Cultivars and perennial germplasm provide the biological context for interpreting molecular mechanisms Table 2. The compendium documents entries for Gunner, Lejinde, and Gongnong No. 6 [17]. Official reports document Zhongcao No. 35, Zhongke No. 1 yellow-flowered(*Medicago falcata*) and variegated materials, and Dongnong 109 [18,19,20]. Registration establishes cultivar identity and an adaptation context; it is not a common-environment ranking of freezing tolerance. The detailed cultivar comparison is retained in Section 6, after the mechanistic and phenotyping evidence.

**Table 2:** Selected alfalfa cultivars with traceable registration or catalogue sources and bounded performance claims.

No.	Cultivar	Record Year	Traceable Record and Performance Boundary	Registered or Intended Region/Use
1	‘Zhongke No. 1’ variegated alfalfa	2024; reported 2025	Regional registration Mengshen-133-2024; institutional notice describes cold adaptation qualitatively [21].	Inner Mongolia registration; local adaptation testing remains necessary [21].
2	‘Gunner’ alfalfa	2022 book	Catalogue entry, p. 322 [17].	—
3	‘Lejinde’ alfalfa	2022 book	Catalogue entry, p. 325; English name is a transliteration [17].	—
4	‘Gongnong No. 6’ variegated alfalfa	2022 book	Catalogue entry, p. 369 [17].	—
5	‘Zhongmu No. 10’ alfalfa	2021	National announcement No. 460 lists the approved cultivar [22].	Huang–Huai–Hai region and comparable environments, as specified in the announcement [22].
6	‘Zhongcao No. 35’ alfalfa	2023	Registration confirmed by an institutional source [18].	Inner Mongolia and similar environments; subject to regional testing [18].
7	‘Jimu No. 1’ alfalfa	2024	Registration reported under Ministry of Agriculture and Rural Affairs announcement No. 820 [23].	—
8	‘Zhongke No. 1’ yellow-flowered alfalfa	2024; reported 2025	Regional registration Mengshen-132-2024; distinct from the variegated cultivar [24].	Inner Mongolia registration; *M. falcata* germplasm context [24].
9	‘Dongnong 109’ alfalfa	2025	National registration Guo S-BV-MS-003-2025. Official description reports winter survival >95% in northern Northeast China [20].	Heilongjiang, Jilin, Liaoning, and eastern Inner Mongolia [20].
10	‘Beilin 203’ alfalfa	2025	Regional registration Mengshen-163-2025, recorded in the issuer’s 2025 annual report [25].	—

Note: Record year denotes the registration year or, for catalogue entries, the source publication year. The 2022 compendium covers cultivars registered during 1987–2020; its publication year is not the registration year of each cultivar. The catalogue locators are Gunner, p. 322; Lejinde, p. 325; and Gongnong No. 6, p. 369 [17]. Only cultivar identities and page locators are summarized for these three entries; Lejinde is a transliteration, not a verified commercial English denomination. An em dash means that no adaptation recommendation is summarized here. The variegated and yellow-flowered Zhongke No. 1 materials are distinct registrations and differ from the 2020 Zhongke No. 1 *M. ruthenica* cultivar [24]. The Dongnong 109 survival percentage is region-specific, not an LT50 or a common-environment comparison [20].

The value of yellow-flowered alfalfa is not the yellow flower color itself. The name commonly denotes the *Medicago falcata* gene pool, a wild or semi-domesticated relative valued for adaptation to cold, drought, and low-fertility environments. Its breeding value lies in alleles and trait combinations that can be introgressed into cultivated M. sativa, while linkage drag, forage quality, yield, and population adaptation must still be evaluated. This germplasm context explains why Mf-prefixed genes are discussed later, while also preventing heterologous validation from being mistaken for direct evidence in cultivated alfalfa.

## 3. Physiological Mechanisms of Cold Tolerance in Alfalfa

### 3.1. ROS Accumulation and Antioxidant Defense

Low temperature can increase ROS production when photosynthetic or respiratory electron flow becomes imbalanced. Chloroplasts and mitochondria are important sources, peroxisomal metabolism produces H_2_O_2_, and plasma-membrane respiratory burst oxidase homologs (RBOHs/NADPH oxidases) generate apoplastic ROS. Their relative contributions depend on organ, illumination, temperature, and acclimation stage. Transient ROS signals can contribute to acclimation, whereas sustained accumulation increases oxidative injury. A rise in antioxidant activity may therefore reflect either effective protection or a response to greater ROS exposure; enzyme activity alone cannot distinguish these alternatives [26,27].

Ca^2+^ and ROS form a reciprocal signaling circuit: cytosolic Ca^2+^ can activate RBOHs, and ROS-sensitive channels can reinforce Ca^2+^ influx. The amplitude, duration, and compartment of this circuit help determine whether ROS acts as a signal or as an injury factor. SOD, CAT, APX, GR, GST, peroxiredoxins, and the ascorbate–glutathione cycle constrain this signal and restore redox homeostasis. Activities of antioxidant enzymes, MDA, H_2_O_2_, O^2−^, and electrolyte leakage are therefore informative readouts, but they should be described as biomarkers unless gene perturbation or rescue links them causally to survival.

Root-crown studies of phosphorus placement, potassium supply, and water–phosphorus combinations connect nutrient management with protective compounds and overwintering performance [28,29,30]. Because responses are dose- and context-dependent, they should be reported against the relevant untreated or alternative-treatment control, not assigned a universal direction. These studies support management-sensitive crown protection, but they do not establish any single antioxidant enzyme or metabolite as the cause of winter hardiness.

Interorganelle coordination adds a further level of regulation. In photosynthetic tissue, chloroplasts, mitochondria, and peroxisomes exchange metabolites through photorespiratory and carbon-metabolic networks, coupling carbon flux with energy and redox balance. Chloroplast- and mitochondrion-derived retrograde signals can also adjust nuclear stress-responsive transcription. Cold-induced perturbation of one compartment may therefore affect the others. This is a mechanistic framework, not proof of a defined interorganelle cold-acclimation circuit in alfalfa: direct flux, compartment-targeted sensor, and perturbation evidence remain scarce, particularly in non-green crowns and overwintering buds. Leaf chloroplast-based models should not be transferred to these organs without testing.

### 3.2. Osmotic Adjustment and Accumulation of Storage Compounds

Osmotic adjustment is a major physiological basis for resistance to cold-induced dehydration in alfalfa. Soluble sugars, sucrose, raffinose, galactinol, proline, soluble proteins, and selected amino acids can lower cellular freezing points, stabilize protein conformation and membrane structure, and improve cellular water retention. Chitosan treatment promotes the accumulation of cold-protective compounds, indicating that exogenous elicitors can serve as auxiliary tools for investigating osmotic adjustment and screening physiological indicators of cold tolerance [31].

Before winter, photosynthates and nitrogenous compounds are redistributed to the root crown and roots, increasing starch, soluble sugars, amino acids, and storage proteins. During acclimation and winter, controlled mobilization of these reserves supports osmotic adjustment and maintenance respiration; after winter, the remaining reserve pool supports bud break and early shoot growth. Consequently, crown reserve concentration should be interpreted together with bud viability, winter survival, spring green-up, and first-cut biomass rather than as an isolated biochemical endpoint [28].

### 3.3. Membrane Stability and Root-Crown Protection

The plasma membrane is both a primary site of cold perception and one of the earliest cellular structures damaged by freezing. Low temperature induces membrane lipid phase transition, decreases membrane fluidity, impairs membrane proteins and ion channels, and disrupts ionic homeostasis. These effects are reflected by increased electrolyte leakage, elevated MDA, and reduced tissue viability. Cold-tolerant materials generally alleviate membrane injury by increasing the proportion of unsaturated fatty acids, strengthening antioxidant defense, accumulating LEA and HSP proteins, and maintaining compatible-solute concentrations.

The root crown is the central organ connecting cold acclimation with perennial persistence. Crown diameter, tissue water status, membrane injury, stored carbohydrate and nitrogen reserves, overwintering-bud number and viability, and vascular integrity influence whether a plant survives winter and resumes growth. A useful organ-to-field sequence is autumn crown acclimation and reserve accumulation → protection of meristems and buds → winter survival → spring green-up and shoot number → first-cut yield. Studies in cold regions should therefore pair leaf measurements with repeated crown and bud observations.

### 3.4. Photosynthesis and Metabolic Regulation

Low temperature inhibits chlorophyll synthesis, photosystem II activity, and electron transport efficiency, thereby reducing chlorophyll concentration, Fv/Fm, effective photochemical efficiency, and net photosynthetic rate, and ultimately constraining carbon assimilation. Cold-tolerant materials generally maintain higher Fv/Fm and a more stable chloroplast structure under low temperature, which supports the supply of storage compounds to the root crown.

Maintenance of carbon assimilation supports the reserve pool available for crown respiration and spring regrowth. Protective sugar accumulation, polyamine turnover, and membrane lipid remodeling can contribute, but their directions need not be identical across organs or acclimation stages. For example, overexpression of MfSAMS1, which encodes S-adenosylmethionine synthetase, increased S-adenosylmethionine and polyamine turnover in tobacco without significantly increasing steady-state polyamine concentrations [32]. This distinction between pool size and metabolic flux helps explain why a metabolite concentration alone is not a sufficient mechanistic endpoint.

## 4. Molecular Regulatory Mechanisms of Cold Tolerance in Alfalfa

The molecular basis of cold tolerance in alfalfa is best viewed as a network connecting perception, compartmented Ca^2+^ and ROS signals, hormonal and circadian regulation, transcription, metabolism, organ protection, and field survival. Gene prefixes indicate source: Ms denotes cultivated *Medicago sativa* and Mf denotes yellow-flowered M. falcata. Evidence from M. truncatula, Arabidopsis thaliana, or Nicotiana tabacum is identified as homologous-model or heterologous evidence and is not treated as direct validation in cultivated alfalfa. Table 3 summarizes each candidate by origin, validation system, pathway, and evidence level.

### 4.1. Ca^2+^ Signaling and Its Links with Sugar Metabolism and ROS Homeostasis

Low temperature can induce Ca^2+^ influx and generate cytosolic signals that are decoded by calmodulin, calmodulin-like proteins, calcium-dependent protein kinases, and CBL–CIPK sensor–kinase modules. Ca^2+^-responsive RBOHs and ROS-sensitive channels provide reciprocal signaling. In cultivated alfalfa, MsCML10 interacts with MsGSTU8, a glutathione S-transferase, and MsFBA6, a fructose-bisphosphate aldolase; this connects Ca^2+^ sensing with ROS control and sugar metabolism [33]. MsCML50 interacts with MsTPP8, a trehalose-6-phosphate phosphatase, linking Ca^2+^ signaling to protective sugar accumulation and redox homeostasis [34]. These experimentally supported modules do not imply that every Ca^2+^–ROS connection has been validated in crowns or field-grown plants.

### 4.2. ICE-CBF/DREB-COR Pathway and Transcription Factor Cascades

**Figure 1:**
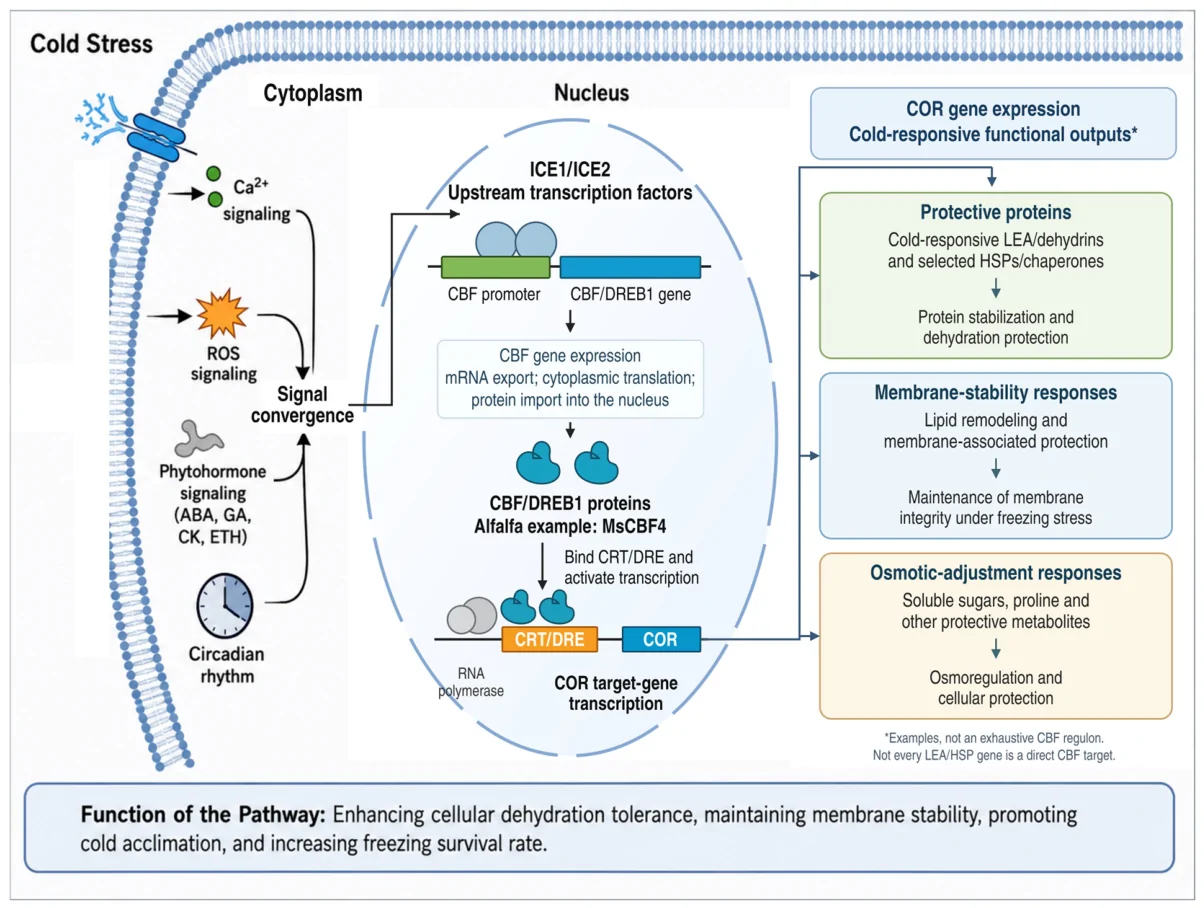
ICE–CBF/DREB–COR core module. The module links cold-responsive upstream signals with CBF/DREB1 expression, CRT/DRE binding, and a subset of protective outputs. Ca^2+^, ROS, phytohormone, and circadian inputs converge on this core, while CBF-independent transcriptional branches operate in parallel; the diagram should therefore be interpreted as a focused module rather than an exhaustive or strictly linear network. Abbreviations: ICE, inducer of CBF expression; CBF/DREB, C-repeat binding factor/dehydration-responsive element-binding; CRT/DRE, C-repeat/dehydration-responsive element; COR, cold-responsive; ABA, abscisic acid; ROS, reactive oxygen species.

The ICE–CBF/DREB–COR pathway is a major cold-acclimation module rather than a single linear route. Cold-regulated ICE activity promotes rapid CBF/DREB1 expression, and CBF proteins bind CRT/DRE elements in a subset of COR genes that contribute to dehydration protection, membrane stabilization, and compatible-solute accumulation. In parallel, CBF-independent branches involving MYB, NAC, WRKY, ERF, and bZIP/ABF factors regulate overlapping and distinct targets. Ca^2+^ and ROS signals, ABA and other hormones, protein modification, and circadian gating determine the timing and amplitude of both branches [35,36]. MsCBF4 provides E2 heterologous support, but direct multi-year validation in cultivated alfalfa remains necessary [37]. Figure 1 therefore represents the CBF core within a broader network, not the complete cold-response system.

### 4.3. ROS Scavenging, Phytohormone Signaling, Autophagy, Circadian Regulation, and Sugar Metabolism

Phytohormones regulate both the threshold and duration of acclimation. ABA perception by PYR/PYL receptors releases SnRK2 kinases from PP2C inhibition, enabling bZIP/ABF-mediated transcription and interaction with sugar, redox, and CBF-related responses. Recent integrated transcriptomic and proteomic evidence in alfalfa implicates the PYL–PP2C–SnRK2–bZIP branch during freezing, while salicylic acid, jasmonate, and ethylene modules contribute context-dependent defense and growth trade-offs [40]. Hormone-associated expression remains candidate evidence unless perturbation is linked to survival in cultivated alfalfa.

Recent direct evidence strengthens the ABA link. MsNCED3 encodes 9-cis-epoxycarotenoid dioxygenase 3, an ABA-biosynthesis enzyme. Its overexpression in cultivated alfalfa increased ABA accumulation and improved cold-response phenotypes, with lower ROS and membrane-injury indicators [52]. Sequential saline–alkali pretreatment and simultaneous combined stress had different outcomes. This E4 result supports ABA-dependent cross-adaptation under the tested regimes, not a recommendation to impose salinity or proof of field winter hardiness.

Autophagy recycles cellular material and can support protein and organelle quality control during stress and energy limitation. MsATG13, which encodes a regulator of autophagy initiation, promoted autophagosome formation, increased antioxidant enzyme activities, and reduced oxidative-injury indicators in transgenic tobacco [38]. This E2 evidence does not establish selective turnover of chloroplasts, mitochondria, or peroxisomes in the alfalfa crown. Measurements of autophagic flux, rather than vesicle abundance alone, should be combined with direct alfalfa perturbation and bud-survival assays to test whether recycling sustains acclimation and regrowth.

Circadian regulation can gate cold-responsive transcription, so temperature responses must be interpreted with sampling time and photoperiod. MsLHY encodes a LATE ELONGATED HYPOCOTYL clock-associated transcription factor. Overexpression and RNA interference in cultivated alfalfa supported its positive contribution to cold tolerance; overexpressing plants showed higher antioxidant activities and lower ROS accumulation and electrolyte leakage under the tested conditions [39]. Whether these effects represent altered phase, amplitude, or downstream cold signaling requires time-series analysis. Crown acclimation, reserve use, and spring regrowth should therefore be assessed across light–dark cycles rather than at one sampling time.

### 4.4. Cold-Tolerance Gene Resources from Yellow-Flowered Alfalfa and Related Species

The *M. falcata* gene pool contributes experimentally tractable candidates, but donor origin must be distinguished from validation host. MfGolS1 encodes galactinol synthase, MfMIPS1 encodes myo-inositol-phosphate synthase, and MfINT-like encodes an inositol transporter-like protein; these candidates connect cold responses with raffinose-family sugars and inositol metabolism [41,42,43]. MfSAMS1, an S-adenosylmethionine synthetase, links signaling with polyamine turnover [32]. MfTIL1 is a temperature-induced lipocalin, and MfPIP2–7 is a plasma-membrane aquaporin [44,45]. MfERF1 and MfNAC3 are transcription factors, MfLEA3 is a late-embryogenesis-abundant protective protein, and MfEF2 is a translation elongation factor. Their functions can be summarized by pathway rather than repeated as enzyme lists. Most evidence comes from tobacco or *M. truncatula* (Table 3); confirmation in tetraploid *M. sativa* crowns and buds remains necessary.

### 4.5. Multi-Omics and Genomic Resources for Resolving Cold-Tolerance Networks

Cultivated alfalfa is an outcrossing autotetraploid with polysomic inheritance, high heterozygosity, and allele dosages from zero to four copies. These features complicate read mapping, haplotype phasing, marker coding, estimation of additive and non-additive effects, and editing of all functional alleles. A marker associated with cold tolerance in one population may also lose predictive value when allele frequency, dosage, linkage phase, or environment changes. Allele-aware reference genomes, diploid and wild-relative genomes, pangenomes, and transgene-free editing platforms now provide the basis for resolving these problems [53,54,55,56].

**Figure 2:**
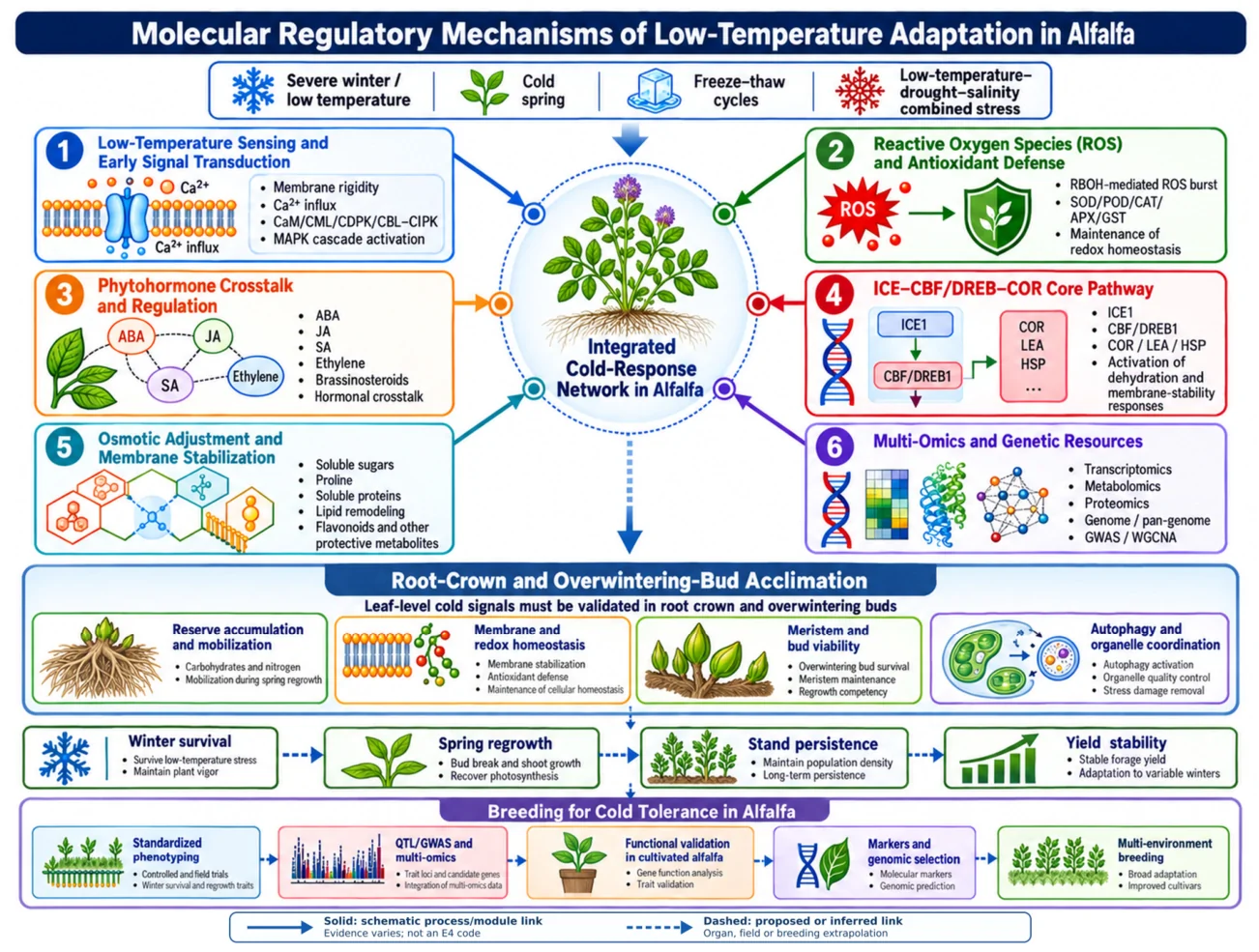
Evidence-aware framework connecting low-temperature perception, Ca^2+^–ROS signaling, hormonal and transcriptional regulation, protective metabolism, and breeding for winter survival. Solid arrows schematically connect processes and modules discussed in the literature, including physiological and multi-omics associations; they do not establish candidate-specific causality or direct physical binding. Dashed arrows mark proposed or inferred connections, including extrapolation to root-crown, overwintering-bud, field, and breeding outcomes. Line style does not encode E1–E5 evidence levels. E4 remains restricted to candidate-specific genetic perturbation in cultivated M. sativa linked to a cold-response phenotype, as defined in Table 3. General mechanisms and heterologous examples retain their stated evidence boundaries. Root-crown reserves and bud viability form the organ-level bridge to spring regrowth and breeding assessment. Candidate sources and evidence definitions are given in Table 3; the breeding workflow represents the proposed application, not a validated causal pathway.

A connected multi-omics pipeline should begin with reliable crown, bud, and field phenotypes and dosage-aware QTL or GWAS signals; overlay transcriptomic, proteomic, metabolomic, and non-coding-RNA responses; use network analysis to prioritize dosage- and organ-consistent candidates; test candidates by direct perturbation in cultivated alfalfa; convert validated variants into marker assays; and evaluate them in breeding populations across environments. Recent work integrating GWAS with whole-transcriptome and ceRNA analysis [57], transcriptomics with proteomics, and root physiology with co-expression networks illustrates the discovery stages of this pipeline. Figure 2 integrates these processes with crown and bud acclimation. Solid arrows schematically connect the literature-discussed response modules; dashed arrows identify proposed links or extrapolation to organ, field, and breeding outcomes. Line style does not encode the E1–E5 evidence levels. Table 3 supplies the candidate-level evidence and validation hosts. Neither a solid arrow nor E4 evidence establishes field winter-survival validation.

## 5. Evaluation Systems for Cold Tolerance in Alfalfa

Cold tolerance is a complex quantitative trait whose meaning depends on scale. Controlled freezing can estimate cellular or whole-plant freezing tolerance, whereas winter hardiness and overwintering survival require field exposure to the winter complex. Evaluation should therefore integrate standardized controlled tests, crown and bud measurements, repeated field observations, and molecular information, while identifying which endpoint each metric predicts.The main evaluation approaches, their indicators, advantages, limitations, and representative evidence are summarized in Table 4.

**Table 4:** Evaluation systems for cold tolerance in alfalfa and their applicable scenarios.

Category	Core Indicators	Advantages	Limitations	Representative Evidence
Field identification	Overwintering survival, green-up rate, cold-injury rating, root-crown injury, overwintering bud number, regrowing shoot number, first-cut yield, and stand cover	Most closely reflects production conditions and perennial stand stability	Strongly affected by year, snow cover, soil water, and field management	Field survival comparisons among cultivars with different dormancy classes provide region-specific winter-performance evidence [58].
Combined field and root assessment	Root activity, root-crown diameter, root-to-shoot ratio, root traits, and winter survival	Links belowground organ traits with field overwintering performance	Requires multi-year, multi-location validation and standardized sampling	Combining root traits with winter survival improved cultivar classification in the Horqin Sandy Land [59].
Physiological and biochemical assessment	Relative electrolyte conductivity, MDA, SOD, POD, CAT, APX, GR, soluble sugars, sucrose, raffinose, proline, soluble proteins, chlorophyll, Fv/Fm, and LT_50_	Quantifies injury and response indicators for controlled prescreening; mechanism requires perturbation	Differences in treatment temperature, sampled organ, and sampling time reduce comparability	Seedlings of cultivars with different fall dormancy levels showed substantial differences in freezing tolerance [60].
Integrated physiological evaluation	Osmotic adjustment, antioxidant enzyme activity, membrane-injury indices, photosynthetic traits, and integrated membership-function analysis	Suitable for comparing cold-tolerance differences among cultivars	Requires calibration against field overwintering performance	Physiological screening of 30 accessions and four-cultivar comparisons separates response profiles; associations require field validation [61,62].
Antioxidant indicator screening	SOD, POD, CAT, APX, MDA, H_2_O_2_, and O^2−^	Rapidly reflects ROS-scavenging capacity	A single enzyme activity cannot represent overall overwintering capacity	Low-temperature treatment of WL-series cultivars showed that SOD, POD, and MDA distinguish antioxidant responses [63].
Molecular identification	CBF/DREB, COR, LEA, HSP, CML, ERF, MYB, WRKY, NAC, bZIP, SNP markers, and omics-derived candidates	Supports early prediction and molecular-assisted selection and connects candidates with breeding applications	Candidates require validation in alfalfa and under field overwintering conditions	QTL mapping with controlled freezing phenotypes identified loci associated with freezing tolerance, survival, and regrowth [64].

Field identification most directly reflects overwintering capacity under production conditions and is suitable for regional trials, cultivar registration, and evaluation before commercial deployment. Physiological and biochemical indicators explain differences in cellular injury, antioxidant defense, osmotic adjustment, and photosynthetic stability and are suitable for germplasm prescreening and mechanistic studies. Molecular identification can improve selection efficiency through candidate-gene expression, molecular markers, QTL mapping, GWAS, WGCNA, transcriptomic modules, metabolic biomarkers, and genomic selection models.

An effective evaluation system should combine multi-year and multi-location winter survival with root-crown injury, bud viability, spring green-up, regrowing shoot number, stand cover, persistence, and first-cut yield. MDA, electrolyte leakage, antioxidant enzyme activity, proline, and related measurements can support screening and mechanism discovery, but their predictive value should be calibrated against survival and regrowth. Molecular markers and omics candidates should be evaluated with independent populations rather than only with the discovery panel.

### 5.1. Standardized Phenotyping and LT50 Determination

Comparable LT50 estimates require explicit reporting of plant age; acclimation temperature, duration, and photoperiod; organ or whole-plant sampling; cooling rate; ice-nucleation temperature and method, or a clear statement that nucleation was not controlled; target temperatures and holding time; thawing conditions; recovery temperature, light, and duration; and biological replication. Temperatures must span both low and high mortality. Chamber air temperature should not be assumed to equal crown or sample temperature: monitoring the tissue or substrate temperature and documenting ice formation improve interpretability. A common parameter set should not be imposed retrospectively on studies using different organs or endpoints.

A concrete controlled-freezing example used intact alfalfa plants acclimated at 4 °C for three weeks, held at 0 °C for 8 h, cooled at 2 °C h^−1^ to −8 °C for 90 min, and rewarmed at the same rate to 2 °C. Recovery was under 23°C/15 °C day/night conditions; survival and regrowth were assessed after two weeks [64]. This was a single-temperature screening assay, not an LT50 curve, and controlled ice inoculation was not specified. LT50 determination would additionally require a graded temperature series and a defined mortality model.

For a mortality-based LT50, the endpoint is death of 50% of the tested plants or explicitly defined crown–bud units after recovery. A fitted binomial logistic model can be written as logit (pdeath) = α + βT, giving LT50 = −α/β; probit models are another option. The temperature–response range, fitted uncertainty, and biological replicate structure should be reported, with an interval estimate and no extrapolated LT50 when 50% mortality is not bracketed. Bud viability, regrowth reduction, visual injury, and electrolyte leakage are distinct endpoints: a fitted 50% injury or response temperature must be named accordingly, unless calibrated against mortality. Raw responses, controls, and recovery criteria are essential for comparing studies.

### 5.2. High-Throughput Phenotyping and Phenomics

High-throughput phenotyping can reduce the cost of repeatedly measuring large perennial populations. UAV-based RGB or multispectral imaging can quantify canopy height, spectral indices, stand cover, autumn growth cessation, snowmelt recovery, and the timing and uniformity of spring green-up. In 210 alfalfa populations, UAV-derived canopy traits supported automated assessment of fall dormancy and forage yield [65], and a 2026 RGB point-cloud pipeline estimated plant height, crown width, and canopy volume in breeding fields [66]. Repeated flights are especially valuable because winter response is a trajectory rather than a single score.

Aboveground remote sensing should be paired with organ-level ground truth. Image-based machine learning has classified root-system architecture from mature field-grown alfalfa roots [67], and related workflows can be adapted to crown diameter, crown cracking, bud number, tissue browning, and root architecture. Phenomics models should be trained and independently validated across years and sites, with destructive crown measurements and survival used to prevent a visually convenient canopy proxy from replacing the causal overwintering organ.

A 2026 Canadian study analyzed 566 fields on 225 farms across four provinces using local conditions, weather, management, and repeated stand observations to model winter-survival risk [68]. Its scale illustrates the value of environment-linked longitudinal phenotypes. The model was not a genomic-selection experiment and does not establish gene-level causality; nevertheless, such environmental metadata can help design representative training populations and independently test the transferability of future genomic predictions.

## 6. Cold-Tolerance Gene Discovery and Progress in Molecular Breeding

Molecular breeding for cold tolerance should connect standardized phenotyping, dosage-aware mapping, evidence-weighted candidate discovery, direct functional validation, marker development, genomic prediction, crossing, and multi-environment selection. The target is an integrated perennial phenotype: crown and bud survival, spring regrowth, stand persistence, yield, forage quality, and adaptation to interacting drought or saline–alkaline stress. Candidate lists or short-term seedling expression alone are insufficient for cultivar development.

### 6.1. Germplasm, Cultivars, and Evidence Translation

Breeding and regional adaptation studies in China have emphasized winter survival, fall dormancy, crown development, and yield stability [69]. Registration, regional adaptation, and mechanistic validation are nevertheless different evidence types. Table 2 summarizes ten cultivar records while distinguishing book entries, national announcements, institutional notices, and an issuer disclosure. Quantitative performance is reported only where the cited source explicitly supplies it, with its geographic boundary. Registration alone does not establish a minimum freezing threshold or a common-environment ranking.

### 6.2. Quantitative Genetics, Genomic Selection, and Genome Editing

QTL and GWAS in autotetraploid alfalfa should retain allele dosage (0–4) and account for polysomic inheritance, relatedness, and population structure. Pseudo-testcross designs using single-dose markers capture only part of segregating variation; a locus near a CBF homolog is not proof that the homolog is causal [64]. High heterozygosity, dosage uncertainty, linkage phase, and structural variation can reduce transferability. Genotype-by-environment interaction may reorder survival rankings across snow cover, soil moisture, freeze–thaw exposure, and management. Crown and field phenotypes across years and locations, followed by independent population validation, are therefore required before marker deployment.

Genomic selection is attractive for winter survival because the phenotype is quantitative, slow, costly, and environment-sensitive. Alfalfa prediction studies establish feasibility for biomass yield and canopy traits, not yet a universal prediction accuracy for winter survival [70,71,72]. Training sets should represent the deployment gene pool, allele-dosage distribution, relatedness, stand ages, and winter environments. Cross-validation should separate families, sites, years, and selection cycles to prevent information leakage. Multi-environment models can include environmental covariates and genotype-by-environment effects; multi-trait indices can balance survival, crown injury, green-up, persistence, yield, and forage quality. Controlled-freezing traits may assist prediction only after calibration against field outcomes. Recalibration with new winters is important when deployment environments differ from the training set.

AtCBF1 transgenic work in cultivated alfalfa is reported in a Master’s thesis, rather than a peer-reviewed research article [73]. This thesis-derived observation is retained with a different documentary status from the peer-reviewed MsCML10, MsCML50, MsLHY, and MsNCED3 studies (Table 3). MsCBF4 and MsATG13 rely on heterologous validation. These results do not yet establish improved winter survival in breeding populations. Genome editing can test candidate causality, but designs must resolve homolog-specific sequence and dosage, account for redundant alleles, and distinguish partial from complete knockouts. Mosaicism, regeneration genotype, unintended edits, and possible growth penalties require evaluation. Stable crown and bud phenotypes and multi-year field performance remain the gate between a functional gene and a deployable breeding target.

International and Chinese programs together provide wild-relative genes, controlled-freezing QTL, high-quality genomes, pangenomes, phenomics, and regional field populations. The priority is to join these resources in a closed pipeline: standardized phenotype → dosage-aware QTL/GWAS → multi-omics and network prioritization → direct alfalfa validation → marker or genomic-prediction model → multi-environment breeding trial → cultivar decision. Recent GWAS–ceRNA, transcriptome–proteome, root-network, combined saline–alkaline–cold, and metabolomic studies broaden the candidate base but remain discovery-stage evidence until they enter this pipeline.The principal breeding routes, representative resources, target traits, and current limitations are summarized in Table 5.

**Table 5:** Progress in cold-tolerance gene discovery and molecular breeding in alfalfa.

Breeding Route	Representative Resources or Genes	Major Methods	Target Traits	Progress and Limitations
Conventional breeding	Local cultivars, bred cultivars, low-fall-dormancy materials, and cold-adapted populations	Field selection, crossing, and multi-year, multi-location assessment of overwintering and yield	Overwintering survival, green-up rate, first-cut yield, and stand persistence	Strong application base in China, but greater integration with molecular markers and genomic selection is needed.
Transgenic validation	AtCBF1 (Master’s thesis), MsCBF4, MsCML10, MsCML50, MsATG13, MsLHY, MsNCED3, and MfERF1	Overexpression, RNAi, heterologous expression, protein-interaction assays, and low-temperature phenotyping	Antioxidant capacity, sugar accumulation, LT_50_, freezing survival, and regrowth	The evidence base is improving, but cultivar development remains constrained by transformation efficiency, genetic stability, and regulation.
Marker and QTL mapping	Freezing-tolerance QTL, CBF-linked loci, and SNP markers	Dosage-aware GBS, linkage mapping, controlled freezing, crown and bud phenotyping, and validation against multi-environment winter survival	Early selection efficiency and selection accuracy	Useful for assisted selection, but requires validation across populations, environments, and years.
GWAS and multi-omics	Transcriptomic modules, metabolic biomarkers, WGCNA candidates, and pan-transcriptomic loci	Dosage-aware GWAS; transcriptomic, proteomic, metabolomic, and ncRNA integration; network analysis; and direct functional validation	Candidate-gene discovery and regulatory-network reconstruction	Suitable for dissecting complex traits, but candidates must be validated in breeding populations.
Genomic selection and editing	Genome-wide SNPs, favorable cold-tolerance alleles, and editable target genes	Training populations matched to the target gene pool; multi-environment and multi-trait prediction; dosage-aware editing; and independent field validation	Coordinated improvement of cold tolerance, yield, quality, and multiple-stress adaptation	High potential for costly winter traits, but allele dosage, polysomic inheritance, heterozygosity, genotype-by-environment interaction, regeneration, and phenotypic data quality remain major constraints.

## 7. Comparison of Research Progress in China and Other Countries

Geographic comparisons are useful for understanding target environments, but the cited studies do not support an exclusive national division of research methods. Chinese programs contribute regional cultivar resources and trials in Northeast China, Inner Mongolia, Northwest China, and cold saline–alkaline environments, alongside functional genetics, genome assemblies, and editing resources. Their regional measurements link crown condition and management with overwintering, while controlled experiments help resolve specific mechanisms.

Complementary examples from other countries include controlled-freezing QTL experiments, alfalfa genomic-prediction studies, image-based root architecture, UAV canopy phenotyping, and Canadian multi-region winter-survival assessment [70,71,72]. These are examples from the cited literature, not a quantitative ranking of national research capacity. Mf-prefixed genes denote *M. falcata* origin, not the country in which a study was conducted; studies of these genes and alfalfa pangenomes should not automatically be classified as research outside China.

The principal opportunity is to connect these complementary resources: shared cold-response definitions, dosage-aware genomic models, repeated crown and bud phenotyping, and independent multi-environment validation. Collaboration is especially valuable where genotypes, management systems, and winter hazards differ. Table 6 therefore summarizes representative contexts and transferable methods without treating them as nationally exclusive approaches.

## 8. Current Challenges and Future Perspectives

Four limitations remain. First, acclimation, cooling, ice nucleation, exposure, recovery, organ choice, and injury criteria are inconsistently reported. Second, expression, biochemical correlation, heterologous transformation, direct alfalfa perturbation, and field validation are often discussed as though they were equivalent. Third, crown and overwintering-bud dynamics from autumn reserve accumulation through spring regrowth remain poorly resolved. Fourth, autotetraploidy, genotype-by-environment interaction, and correlated responses in yield, quality, persistence, drought, and saline–alkaline tolerance complicate translation into cultivars.

Future research should treat the root crown–overwintering bud unit as the primary organ-level target, rather than using leaf responses alone as a substitute for perennial survival. Standardized freezing assays should be paired with repeated crown injury, bud viability, winter survival, spring green-up, shoot number, persistence, and first-cut yield. Time-resolved sampling should measure organelle redox state, membrane lipids, carbohydrate and nitrogen reserves, ABA, autophagic flux, and circadian phase. Direct perturbation in cultivated alfalfa should prioritize candidates supported across organs, omics layers, allele dosage, and contrasting genotypes.

Breeding pipelines should integrate UAV and ground-based phenomics, QTL and GWAS, pangenomes, transcriptomics, proteomics, metabolomics, non-coding RNA, network analysis, genome editing, and genomic selection with conventional crossing. Training populations and prediction models should represent target environments and combined stresses; recent saline–alkaline–cold and metabolomic studies illustrate why selection under cold alone may miss production-relevant responses [74,75]. The practical endpoint is a cultivar whose crown and buds survive winter and whose stand regrows uniformly while maintaining yield and forage quality across environments.

## Figures and Tables

**Table 3:** Reported cold-tolerance-related genes or modules in alfalfa and related species, classified by validation system and evidence level.

No.	Gene or Module	Origin	Validation System and Evidence Level	Pathway	Functional Evidence	Ref.
1	MsCML10-MsGSTU8/MsFBA6	*M. sativa*	E4—cultivated *M. sativa*, *M. truncatula*, and interaction assays	Ca^2+^ signaling, ROS scavenging, and sugar metabolism	CML10 activates glutathione S-transferase GSTU8 and aldolase FBA6; genetic and biochemical assays link ROS reduction and sugar accumulation to cold tolerance.	[33]
2	MsCML50-MsTPP8	*M. sativa*	E4—overexpression and RNAi in cultivated *M. sativa*	Ca^2+^ signaling, trehalose metabolism, and ROS homeostasis	CML50 activates trehalose-6-phosphate phosphatase TPP8; protective sugars and proline accumulate and antioxidant capacity increases.	[34]
3	CBF signaling network	General plant system	General-plant synthesis; no candidate-specific alfalfa grade	ICE-CBF/DREB-COR	The CBF network links cold perception and protein modification with COR gene expression and functions as a central cold-acclimation module.	[35]
4	C-repeat binding factor regulatory module	General plant system	General-plant synthesis; no candidate-specific alfalfa grade	CBF/DREB transcriptional regulation	CBFs are regulated by Ca^2+^, ROS, phytohormones, circadian rhythms, and other signals.	[36]
5	MsCBF4 (CBF/DREB transcription factor)	*M. sativa*	E2—*M. sativa* gene expressed in *A. thaliana*	ICE-CBF/DREB-COR	Expression is induced by low temperature and freezing, and overexpression improves growth and survival under frost.	[37]
6	MsATG13 (autophagy regulator)	*M. sativa*	E2—*M. sativa* gene expressed in *N. tabacum*	Autophagy and antioxidant regulation	Promotes autophagosome formation, increases SOD, POD, and CAT activity, and decreases H_2_O_2_, O^2−^, and MDA.	[38]
7	MsLHY (circadian clock factor)	*M. sativa*	E4—overexpression and RNA interference in cultivated *M. sativa*	Circadian regulation and low-temperature response	Supports cold tolerance; overexpression increases antioxidant activities and decreases ROS and electrolyte leakage.	[39]
8	SA defense module	*M. sativa*	E3—SA treatment and transcriptomics in *M. sativa*	SA signaling, MAPK/WRKY regulation, and antioxidant defense	SA treatment improves freezing-response phenotypes; defense and redox transcripts provide candidates, not validation of every inferred gene or pathway.	[40]
9	MfGolS1 (galactinol synthase)	*M. falcata*	E2—*M. falcata* gene expressed in *N. tabacum*	Galactinol and raffinose metabolism	Promotes accumulation of protective sugars and improves tolerance to low temperature and multiple stresses.	[41]
10	MfMIPS1 (myo-inositol-phosphate synthase)	*M. falcata*	E2—*M. falcata* gene expressed in *N. tabacum*	Myo-inositol synthesis	Induced by low temperature, H_2_O_2_, and NO and improves tolerance to low temperature and dehydration.	[42]
11	MfINT-like (inositol transporter-like)	*M. falcata*	E2—*M. falcata* gene expressed in *N. tabacum*	Myo-inositol transport	Participates in inositol transport and cold-induced sugar metabolism and improves low-temperature adaptation.	[43]
12	MfSAMS1 (S-adenosylmethionine synthetase)	*M. falcata*	E2—*M. falcata* physiology and transgenic *N. tabacum*	ABA, H_2_O_2_, NO, and polyamine metabolism	Increases SAM and polyamine synthesis/oxidation in tobacco; steady-state polyamine levels need not increase.	[32]
13	MfTIL1 (temperature-induced lipocalin)	*M. falcata*	E2—*M. falcata* gene expressed in *N. tabacum*	Lipid protection and oxidative-stress defense	Encodes a temperature-induced lipocalin and increases tolerance to low temperature and oxidative stress.	[44]
14	MfPIP2–7 (aquaporin)	*M. falcata*	E2—*M. falcata* gene expressed in *N. tabacum*	Aquaporin-mediated cold response	Improves growth under low temperature, freezing, and nitrate deficiency.	[45]
15	MfERF1 (ethylene-response factor)	*M. falcata*	E3—*N. tabacum* overexpression plus *M. truncatula* interference	Ethylene response, polyamines, and antioxidant defense	Improves cold tolerance through polyamine turnover, antioxidant protection, and proline accumulation.	[46]
16	MfNAC3 (NAC transcription factor)	*M. falcata*	E3—overexpression in *M. truncatula*	NAC transcriptional regulation	Enhances low-temperature tolerance in *Medicago truncatula*.	[47]
17	MfLEA3 (LEA protective protein)	*M. falcata*	E2—*M. falcata* gene expressed in *N. tabacum*	LEA protective protein	Induced by cold, dehydration, and ABA and improves tolerance to low temperature, drought, and high light.	[48]
18	MfEF2 (translation elongation factor)	*M. falcata*	E2—*M. falcata* gene expressed in *N. tabacum*	Translation elongation and photosynthetic protection	Reduces ROS accumulation after low temperature and improves photosynthetic efficiency and freezing survival.	[49]
19	Ms/Mf transcription factor groups	*M. sativa* and *M. falcata*	E1—cold-treatment transcriptomics in *M. sativa* and *M. falcata*	MYB, NAC, bZIP, WRKY, and ERF	Provides candidate low-temperature-responsive transcription factors and targets for molecular-marker development.	[50]
20	FIB genes (fibrillin candidates)	*M. sativa* populations	E1—pan-transcriptome association in *M. sativa* populations	Putative plastid lipid-associated fibrillins; cold-response co-expression	Downregulation of FIB genes is associated with the cold-stress response. Plastid localization and a causal tolerance function remain unvalidated in alfalfa.	[51]
21	MsNCED3 (ABA-biosynthesis enzyme)	*M. sativa*	E4—overexpression in cultivated *M. sativa*	ABA synthesis and stress cross-adaptation	Increases ABA; improves cold-response phenotypes under tested regimes. Crown and field validation remain open.	[52]

Note: E1, expression, co-expression, association, or omics evidence without functional perturbation; E2, functional validation in a non-*Medicago* heterologous host; E3, functional evidence in a *Medicago* model, or organ/physiological-treatment evidence without candidate-specific genetic perturbation in cultivated alfalfa; E4, candidate-specific genetic perturbation in cultivated *M. sativa* linked to a cold-response phenotype; E5, replicated candidate/allele validation for winter survival in multi-year field trials or breeding populations. Interaction assays support the mechanism but do not alone establish E4. These categories describe evidence context, not a universal ranking of experimental quality; the host and organ must also be considered. General-plant pathway reviews are not assigned candidate-specific levels. None of the listed candidates has E5 evidence for alfalfa winter survival.

**Table 6:** Representative research contexts in China and other countries and opportunities for integration.

Dimension	Examples from China	Complementary Examples from other Countries	Implications for Future Research in China
Germplasm utilization	Local cultivars, bred cultivars, yellow-flowered and variegated alfalfa, cultivars grown in cold regions, and regional trial materials; strong emphasis on natural overwintering and regional adaptation	Mapping and recurrent-selection populations; Canadian multi-region farm/field observations. These resources complement regional cultivar trials [64,68].	Establish a core cold-tolerance germplasm collection and introgress favorable alleles from wild relatives into cultivated populations.
Evaluation systems	Frequent use of overwintering survival, green-up rate, cold-injury rating, root-crown traits, and physiological and biochemical indicators that closely reflect production conditions	Controlled-freezing screens, root image analysis, and UAV canopy phenotyping; these methods are not geographically exclusive [64,65,67].	Standardize acclimation, cooling, ice nucleation, recovery, and endpoints; add repeated UAV green-up and stand-persistence measures; and retain root-crown ground truth.
Molecular mechanisms	Functional studies of MsCML10, MsCML50, MsATG13, MsLHY, and MsNCED3, together with wild-relative genes and genomic resources [32,33,34,38,39,41,42,43,44,45,46,47,48,49,50,51,52,53,54,55,56].	Physiological and genetic study of alfalfa–rhizobium associations under simulated overwintering; comparison of tissue and whole-plant responses [5].	Strengthen transformation in alfalfa, dynamic sampling of the root crown, and validation under natural overwintering conditions.
Molecular breeding applications	Strong progress in cultivar registration and conventional breeding, whereas molecular-marker and genomic-selection applications remain limited	Alfalfa genomic prediction for yield and canopy traits, and controlled-freezing QTL as candidate tools for winter-survival breeding [64,70,71,72].	Establish a dosage-aware pipeline linking phenotypes, multi-omics, direct alfalfa validation, markers, genomic prediction, and multi-environment breeding populations.

## Data Availability

No new datasets were generated for this narrative review. The evidence discussed is available in the cited publications and cultivar sources.

## Abbreviations

The following abbreviations are used in this manuscript:

Ca^2+^	calcium ion
APX	ascorbate peroxidase
CAT	catalase
CBF	C-repeat binding factor
CML	calmodulin-like
COR	cold-responsive
FBA	fructose-bisphosphate aldolase
GR	glutathione reductase
HSP	heat-shock protein
ICE	inducer of CBF expression
LEA	late embryogenesis abundant
LT50	temperature causing 50% mortality
MDA	malondialdehyde
POD	peroxidase
ROS	reactive oxygen species
SOD	superoxide dismutase
TPP	trehalose-6-phosphate phosphatase
ABA	abscisic acid
ceRNA	competing endogenous RNA
G×E	genotype-by-environment interaction
GWAS	genome-wide association study
HTP	high-throughput phenotyping
ncRNA	non-coding RNA
PP2C	protein phosphatase 2C
QTL	quantitative trait locus
RBOH	respiratory burst oxidase homolog
RGB	red–green–blue
SnRK2	SNF1-related protein kinase 2
UAV	unmanned aerial vehicle
WGCNA	weighted gene co-expression network analysis
ABF	ABA-responsive element-binding factor
bZIP	basic leucine zipper
CaM	calmodulin
CBL	calcineurin B-like protein
CDPK	calcium-dependent protein kinase
CIPK	CBL-interacting protein kinase
DREB	dehydration-responsive element-binding factor
ERF	ethylene-responsive factor
Fv/Fm	maximum quantum efficiency of photosystem II
GBS	genotyping-by-sequencing
GS	genomic selection
GST	glutathione S-transferase
JA	jasmonic acid
MAPK	mitogen-activated protein kinase
NAC	NAM, ATAF1/2, and CUC2 transcription-factor family
NCED	9-cis-epoxycarotenoid dioxygenase
NO	nitric oxide
PYR/PYL	PYRABACTIN RESISTANCE/PYRABACTIN RESISTANCE-LIKE ABA receptors
SA	salicylic acid
SAM	S-adenosylmethionine
SNP	single-nucleotide polymorphism
